# An Integrated B_1_^+^ Efficient Triple‐Tuned (^2^H/^23^Na/^31^P) Body Coil at 7T

**DOI:** 10.1002/nbm.70118

**Published:** 2025-08-07

**Authors:** Busra Kahraman‐Agir, Jiying Dai, Dimitri Welting, Martijn Lunenburg, Mark Gosselink, Dennis Klomp

**Affiliations:** ^1^ Department of Radiology University Medical Center Utrecht Utrecht the Netherlands; ^2^ Tesla Dynamic Coils Zaltbommel the Netherlands

**Keywords:** birdcage efficiency, loss mechanism, tank (trap) circuits, triple‐tuned body coil

## Abstract

The electrical loss mechanisms of multi‐tuned birdcages were studied to design an efficient triple‐tuned (^2^H/^23^Na/^31^P) body coil at 7T. The sources of RF power loss were investigated in three aspects, namely, resistive losses in copper conductors, dielectric losses of capacitors, and resonance‐induced losses in tank circuits of multi‐tuned circuits all normalized to the intrinsic losses in tissue of a single‐tuned birdcage. A 24‐leg triple‐tuned (^2^H/^23^Na/^31^P) high‐pass birdcage coil with two out of three short circuits in the end rings (i.e., eight effective legs) connected to tuning capacitors (16caps‐birdcage) was compared with a classical 24‐leg double‐tuned (^2^H/^31^P) birdcage coil (48caps‐birdcage). The electric loss in the tissue remains dominant over the coil conductor loss. When loaded with the head in the center and copper and capacitor losses were included, decreasing the number of legs in the birdcage design can increase the B_1_^+^ field intensity by 6%–41% at the frequency range from ^31^P to ^2^H. The loss factor of capacitors is amplified when incorporating multi‐tuning circuitries. That is, the dominant non‐tissue losses are caused by the multi‐tuned circuits as they contribute to a 67%/60%/49% decrease in the B_1_^+^ field intensity when considering a 24‐leg design at ^2^H/^23^Na/^31^P frequencies, respectively. Therefore, the triple‐tuned wide‐leg 16caps‐birdcage experimental design employing 16 triple‐tuned circuits maintained or even improved the coil efficiency by 0 dB/3.45/3.75 dB at ^2^H/^23^Na/^31^P frequencies compared with the classical 24‐leg design utilizing 48 double‐tuned circuits. The B_1_^+^ maps of the triple‐tuned wide‐leg 16caps‐birdcage design showed 5.1, 4.1, and 13.5 μT of B_1_^+^ field intensity at the center of the body phantom at ^2^H, ^23^Na, and ^31^P frequencies, respectively. Reducing the number of effective legs in a triple‐tuned birdcage is an impactful method to maintain relatively high B_1_^+^ efficiency.

## Graphical abstract

The high loss of the body and multi‐tuned circuits is dominant in birdcage coils. Reducing the number of effective legs to eight in a 24‐leg triple‐tuned body coil maintained or even improved the coil efficiency by 0 dB/3.45/3.75 dB at ^2^H/^23^Na/^31^P frequencies compared with the classical 24‐leg design utilizing 48 double‐tuned circuits.

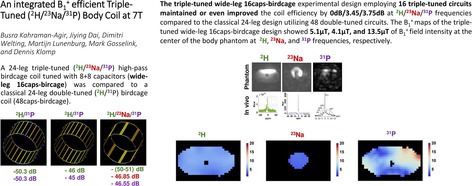

**Abbreviations**
^2^H: deuterium
^23^Na: sodium
^31^P: phosphorus
FID: free induction decay
MRI: magnetic resonance imaging
MRSI: magnetic resonance spectroscopic imaging
RF: radiofrequency
SAR: specific absorption rate
UHF: ultra‐high field

## 1 Introduction

The birdcage coil geometry [1] is most often utilized in the implementation of transmit radiofrequency (RF) coils owing to its distinctive features yielding a homogeneous magnetic field distribution across a substantial portion of the coil volume. Additionally, this geometry results in a non‐zero B_1_^+^ intensity at the center, leading to exceptional volume excitation capabilities. The primary focus for the birdcage geometry is to ensure homogeneity and to optimize its efficiency by adjusting the coil parameters. For instance, implementing a high‐pass geometry for a single‐tuned small‐volume birdcage can yield high B_1_^+^ intensity at ultra‐high field (UHF) MRI [2]; employing shorter coil length on a single‐tuned birdcage can improve the axial coil efficiency [1, 3]; and increasing the number of legs to eight or more can enhance homogeneity [4]. Nevertheless, such optimization methods can only be effective to some extent considering the dominant loss effect of high‐power capacitors [5, 6].

X‐nuclei MRSI, in other words, metabolic imaging, has maintained its importance over the past four decades. While ^31^P MRS provides insights into phosphate metabolites and cellular energetics [7], ^23^Na MRI is employed to evaluate the physiology of the brain, breast, muscles, and cancer metabolism [8]. Alternatively, ^2^H MRS contributes to the understanding of glucose metabolism [9]. On the other hand, a major challenge in metabolic image acquisition lies in the low abundance of these nuclei in the body and the unavailability of state‐of‐the‐art RF coil setups optimized for these nuclei. Therefore, dedicated integrated hardware is required both for transmit and receive purposes for X‐nuclei MRI. Consequently, this fact has sparked growing interest in the field of multi‐tuned RF coils. Especially, double‐tuned birdcage designs have gained prominence since the 1990s. Various methodologies have been proposed for designing such coils, including: (1) incorporating passive elements [10, 11, 12, 13, 14, 15, 16], (2) employing active switches [17, 18], (3) utilizing a four‐ring approach [19, 20], (4) applying geometrical decoupling [21], and (5) using metamaterial‐based structures [22].

When using the passive elements, most double‐tuned birdcages include one or more tank circuits. These double‐tuned circuits are placed on the typical location of the tuning capacitors in a conventional birdcage design. Consequently, the number of double‐tuned circuits is equal to the number of originally used tuning capacitors. However, Schnall et al. [15] emphasized the significance of tank circuit losses [10, 13, 23, 24, 25]. Hence, increasing the number of legs to enhance the homogeneity of a multi‐tuned birdcage design is in conflict with improving its efficiency. Additionally, recent studies have highlighted the significant dominance of tissue noise over coil noise at UHF MRI [23, 24]. Therefore, conducting a comprehensive examination at UHF to delve into the loss mechanisms in multi‐tuned birdcages is crucial to pave the way for an efficient B_1_^+^ field.

Our goal in this study is to understand the relative contribution of loss sources from conductors, capacitors, double‐tuned circuits, and triple‐tuned circuits in optimizing the B_1_^+^ field strength to design the first triple‐tuned (^2^H/^23^Na/^31^P) integrated efficient birdcage body coil at 7T. First, the loss of capacitors was examined extensively by verifying their specifications in the experimental condition [6]. Then, double‐tuned and triple‐tuned circuits were created in a circuit simulation environment to calculate their effective resistance by including the Q values of each component. Afterwards, a double‐tuned loop coil and a triple‐tuned loop coil were built on the bench using the same components as in the simulations. Q values of the loop coils were measured to indirectly calculate the loss of the double‐tuned and triple‐tuned circuits in the experimental setup. Then, two 24‐leg birdcage coils having the same dimension were created in an electromagnetic (EM) simulation environment such that one design had 2 × 8 short circuits on each end ring (less effective legs), resulting in fewer tuning capacitors (16caps‐birdcage) than the other (48caps‐birdcage). The B_1_^+^ field strength of the birdcage coils was calculated in the presence of capacitor loss and multi‐tuned loss. The double‐tuned 16caps‐birdcage and the triple‐tuned 16caps‐birdcage coils were built on the bench to verify the simulation results by bench results. The validity of our approach is substantiated through phantom and in vivo studies.

## 2 Methods

Experiments were conducted considering Larmor frequencies of ^2^H, ^23^Na, and ^31^P at 7T (Philips, Best, the Netherlands), which are 45.75, 78.80, and 120.68 MHz, respectively.

### 2.1 Loss Calculation

#### 2.1.1 The Copper Conductor Loss

The investigation of the loss mechanisms of the copper conductors of a 40‐cm‐long 24‐leg high‐pass birdcage coil (Ø = 60 cm) concentrated on two aspects: the loss of one leg and the loss of an endring piece positioned between two tuning capacitors. The length of a single leg and each endring piece was 40 and 7 cm (≅60π/24), respectively. Two loop coils with a 40 cm long conductor (L40) and a 7 cm long conductor (L7) were created to indirectly compute the loss in a single leg (R_leg_) and a single endring piece (R_endring_), respectively. L40 and L7 were tuned using small‐case capacitors characterized by negligible losses (100B, AVX, USA) at ^2^H, ^23^Na, and ^31^P frequencies. The quality factors (Q) of L40 and L7 were measured across all three frequencies, and their corresponding losses were subsequently calculated. Notably, all copper conductors employed in both the birdcage and the loops were standardized at a width of 1 cm.

#### 2.1.2 Capacitor Loss

In the construction of transmit coils, large‐case capacitors (100E, AVX, USA) were selected for their durability under high voltage conditions. As outlined in the specification document of the 100E series [6], the Q‐factors for this capacitor series at 30 MHz served as a basis for calculating losses at the ^2^H and ^23^Na frequencies.

Capacitor losses at the ^31^P frequency were assessed by taking the specification document into account and by conducting bench experiments. Initially, an approximate line representing Q‐factors at 150 MHz was estimated, referring to the Q‐factor trends of the 100C series [25]. Knowing the Q‐factors, the loss can be derived by $\frac{1}{\left(2\text{πfQC}\right)}$, where *f* is the operating frequency, and *C* is the capacitance. The calculated results were validated at a few sampled capacitance values by on‐bench experiments. Two loop coils, a 13 cm long coil (L13) and a 38 cm long coil (L38), were tuned at the ^31^P frequency using various methods, such as serial and parallel capacitor connections, in order to include more capacitance values in the validation as possible. For instance, L13 was tuned by a single serial 30 pF capacitor and alternatively, by three 10 pF capacitors connected in parallel. Similarly, L38 was tuned using a single serial 10 pF capacitor and three serial 30 pF capacitors. The corresponding Q values were measured for each configuration. In the process of calculating losses, the equivalent circuitry for each case was considered, resulting in the final loss values being derived through division and multiplication based on the number of elements in the serial and parallel connections, respectively. The loss of each capacitor was then calculated accordingly.

#### 2.1.3 The Loss of the Double‐Tuning Circuit

**FIGURE 1:**
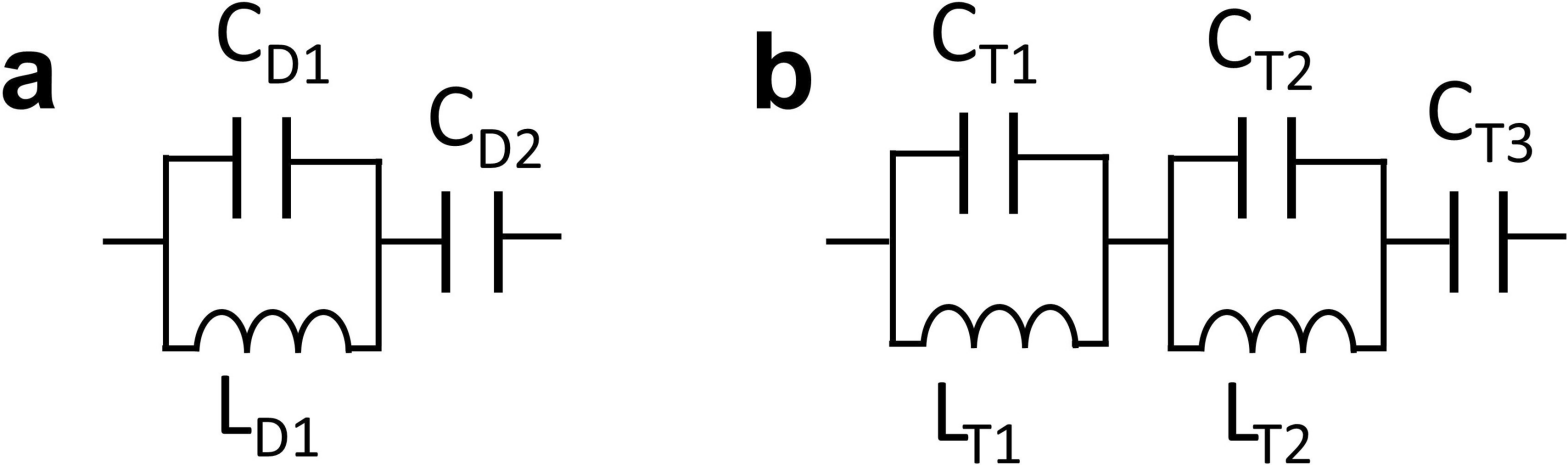
(a) The double‐tuned circuit. (b) The triple‐tuned circuit. (c) The simulated 24‐leg high pass birdcage coil (48caps‐birdcage) where the loss of each leg, the loss of the endring piece, and the loss of the tuning capacitor are represented by R_leg_48_, R_endring_48_, and R_cap_48_. (d) The simulated 24‐leg high pass birdcage coil tuned by short circuiting two out of three end ring capacitors (16caps‐birdcage) where the loss of each leg, the loss of the endring piece, and the loss of the tuning capacitor are represented by R_leg_16_, R_endring_16_, and R_cap_16_.

A tank circuit and a serial capacitor (Figure 1a) were implemented [15] as a double‐tuned circuit configuration. Specifically, two configurations were devised: a ^2^H/^31^P double‐tuned and a ^23^Na/^31^P double‐tuned circuit. The loss of the double‐tuned circuit was evaluated by an indirect approach, where the double‐tuned circuit was soldered onto a 25‐cm‐long loop coil. The loop coil's Q‐factors at the corresponding frequencies were measured. Afterwards, measured Q‐factors of the ^2^H/^31^P double‐tuned and the ^23^Na/^31^P double‐tuned circuits were used to compute losses for all three frequencies.

#### 2.1.4 The Loss of the Triple‐Tuning Circuit

A triple‐tuned circuit configuration was implemented, employing two serial tank circuits and a serial capacitor [15] (Figure 1b). The loss of the triple‐tuned circuit was indirectly assessed by soldering onto a 25‐cm‐long loop coil and measuring the Q‐factors. The Q‐factors of the ^2^H/^23^Na/^31^P triple‐tuned circuit were measured for all nuclei, and subsequent calculations were performed to evaluate the losses.

### 2.2 Simulation Studies

#### 2.2.1 Circuit Simulations

Two double‐tuned circuits (^2^H/^31^P, ^23^Na/^31^P) and a triple‐tuned circuit (^2^H/^23^Na/^31^P) were created in a circuit simulation environment (AWR, Cadence Design Systems). The components for the double‐tuned ^2^H/^31^P circuit were C_D1_ = 56 pF, C_D2_ = 33 pF, L_D1_ = 68 nH; for the double‐tuned ^23^Na/^31^P circuit, they were C_D1_ = 20 pF, C_D2_ = 68 pF, L_D1_ = 23 nH; and for the triple‐tuned ^2^H/^23^Na/^31^P circuit, they were C_T1_ = 68 pF, C_T2_ = 47 pF, C_T3_ = 24 pF, L_T1_ = 39 nH, L_T2_ = 100 nH. The loss of the components was included in the simulation by considering their Q values. The real part of the impedance of the circuits was used to calculate the circuit loss.

#### 2.2.2 EM Simulations

A 40‐cm‐long 24‐leg high‐pass birdcage coil (Ø = 59 cm) alongside a shield (Ø = 63.5 cm) was created in a 3D EM simulation software (Sim4life, Zurich MedTech AG, Switzerland). Both the birdcage coil and the shield were modeled as perfect electric conductors (PECs). Maintaining consistent coil parameters, including the number of legs, the length, and the diameter, a comparative study was facilitated between two distinct birdcage designs: the 48caps‐birdcage and the 16caps‐birdcage.

**FIGURE 2:**
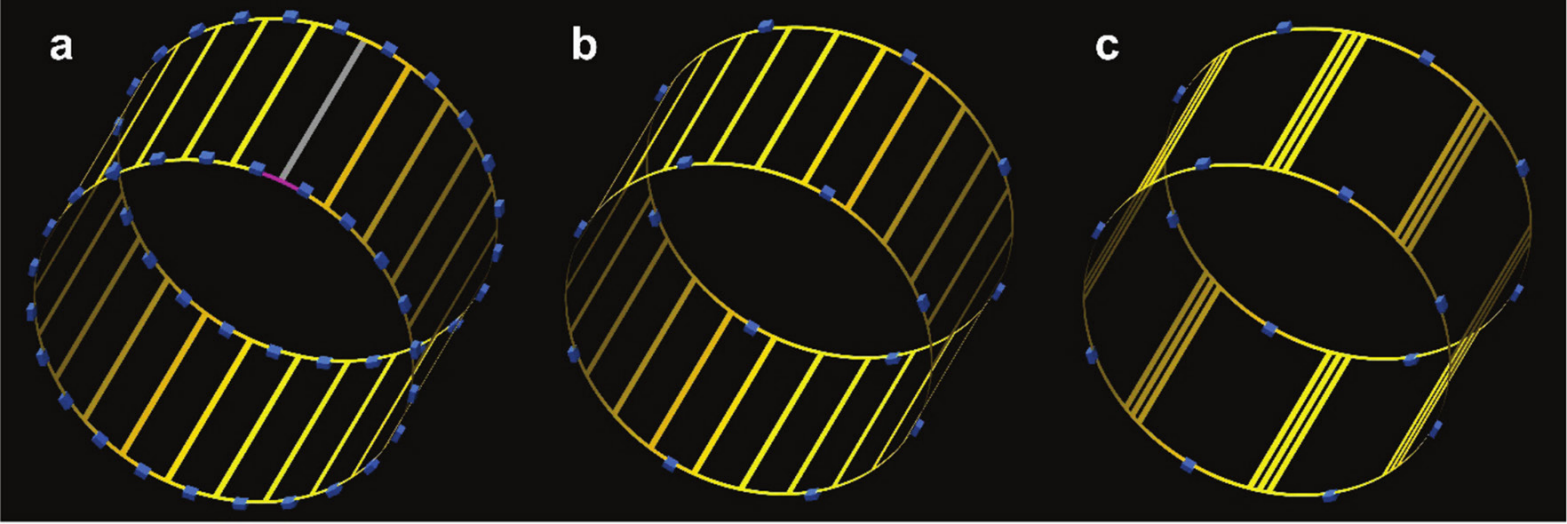
Three different configurations of a 24‐leg high‐pass birdcage (Ø = 60 cm, length = 40 cm) where the blue squares are tuning capacitors. (a) The 48caps‐birdcage design which is tuned classically by 24 + 24 capacitors. The pink and the gray parts indicate the conductor piece used in calculating the loss of a 7‐cm‐long endring piece (R_endring_) and a 40‐cm‐long leg (R_leg_). (b) The 16caps‐birdcage design which is tuned by 8 + 8 capacitors by shorting every two out of three legs. (c) The wide‐leg 16caps‐birdcage design where every three legs are placed closely and the angle between every leg group is 45°.

The 48caps‐birdcage design was tuned classically as a high‐pass birdcage; that is, employing a total of 48 tuning capacitors (Figure 2a), 24 for each endring. Alternatively, the 16caps‐birdcage design followed a novel approach by short‐circuiting two out of every three capacitor gaps on the endrings (Figure 2b). This design modification resulted in the utilization of eight tuning capacitors per endring, totaling 16 tuning capacitors.

B_1_^+^ field intensity was subsequently computed under two distinct loss cases, namely, (1) R_leg_, R_endring_, and R_cap_ included and (2) R_leg_, R_endring_, and R_MT_. R_leg_, R_endring_, and R_cap_ were determined by the aforementioned calculations and bench measurements. In the R_leg_, R_endring_, and R_MT_ case, R_leg_ was connected in serial to each leg; R_endring_ and R_MT_ were connected in serial to the endrings. R_MT_ is the loss of the multi‐tuning circuit.

Furthermore, diverse loading cases were explored, namely, the human model's [26] head, the human model's abdomen, and a body phantom (26.13 L, *σ* = 0.55 S/m, *ε* = 74). These investigations sought to elucidate the impact of loading types on the B_1_^+^ field efficiency. The abdomen and phantom cases are considered as the load loss‐dominated cases, whereas the head case is referred to as the coil loss‐dominated case. In addition, local SAR for each coil was computed.

### 2.3 Birdcage Designs On‐Bench

We designed two different high‐pass birdcages: the 48caps‐birdcage (Figure 2a) and the 16caps‐birdcage. The 16caps‐birdcage was designed in two versions: either by short‐circuiting two out of three endring segments (Figures 2b and S1b), or by moving the short‐circuited legs close to each other to effectively act like an 8‐leg (wide‐leg) birdcage (Figures 2c and S1c,d). The parameters of the birdcages, including length (40 cm), diameter (60 cm), and conductor width (1 cm), were maintained the same across all designs. The right mode of each birdcage was determined by two pick‐up probes and by observing a stable peak on the S_21_ graph when moving the probe from one side to the other side in the birdcage. The coupling between a matched birdcage port and a pick‐up probe refers to coil efficiency. This parameter is monitored via S_21_. To plot S_21_, the birdcage port is connected to channel‐1 of a VNA (Vector Network Analyzer, Copper Mountain Technologies, USA) and a pick‐up probe connected to channel‐2 of the VNA. The same pick‐up probe was used in all coil efficiency measurements and rotated slightly around the center of the FOV to search for the maximum S_21_. A body phantom (26.13 L, *σ* = 0.55 S/m, *ε* = 74 at 120 MHz) with a hole in the center that fits the pick‐up probe was employed to load the birdcage coils during the matching process and the efficiency measurements on the bench. S_11_2H_, S_11_23Na_, and S_11_31P_ refer to the reflection coefficient of Port 1 of the birdcage coil for ^2^H, ^23^Na, and ^31^P frequencies, respectively. S_22_2H_, S_22_23Na_, and S_22_31P_ refer to the reflection coefficient of Port 2 of the birdcage coil for ^2^H, ^23^Na, and ^31^P frequencies, respectively.

#### 2.3.1 48caps‐Birdcage

A 24‐leg double‐tuned ^2^H/^31^P 48caps‐birdcage (Figure S1a) is our default operating transmit bore coil in our 7T system (Achieva with DDAS upgrade, Philips, Best, the Netherlands). This birdcage coil is referred to as the reference double‐tuned body coil throughout the text. Forty‐eight double‐tuned circuits (Figure 1a, C_D1_ = 100 pF, L_D1_ = 30 nH, C_D2_ = 159 pF) in total were employed in the reference double‐tuned body coil. The efficiency of the reference double‐tuned body coil was measured with the body phantom.

#### 2.3.2 16caps‐Birdcage

Several versions of the 16caps‐birdcage were designed, simulated, and constructed to assess multiple single‐tuned (^2^H, ^23^Na, and ^31^P), multiple double‐tuned (^2^H/^31^P, ^23^Na/^31^P) and a triple‐tuned (^2^H/^23^Na/^31^P) setup all with effectively 8 × 3 parallel rods and 16 tuning circuits (see Figure S2 for full overview) [27]. The components for the single‐tuned ^2^H, ^23^Na, and ^31^P were 100, 30, and 11 pF, respectively. For the double‐tuned ^2^H/^31^P and ^23^Na/^31^P circuit components (Figure 1a) were C_D1_ = 56 pF, L_D1_ = 68 nH, C_D2_ = 33 pF, and C_D1_ = 68 pF, L_D1_ = 23 nH, C_D2_ = 20 pF, respectively. For the triple‐tuned ^2^H/^23^Na/^31^P circuit components (Figure 1b) were C_T1_ = 68 pF, C_T2_ = 47 pF, C_T3_ = 24 pF, L_T1_ = 39 nH, and L_T2_ = 100 nH. Afterwards, the efficiency of the single‐tuned, double‐tuned, and triple‐tuned versions was measured when loaded and matched. Additionally, another triple‐tuned (^2^H/^23^Na/^31^P) wide‐leg 16caps‐birdcage coil equipped with a segmented (to minimize gradient field induced eddy‐currents) RF shield (Figure S1c) was constructed on a mechanical former that could be shifted inside the bore of the 7T MRI for basic phantom and in vivo experiments. The triple‐tuned (^2^H/^23^Na/^31^P) wide‐leg 16caps‐birdcage was matched via triple‐matching (Figure S3).

### 2.4 Phantom and In Vivo Imaging

The triple‐tuned (^2^H/^23^Na/^31^P) wide‐leg 16caps‐birdcage coil (Figure S1c) with the segmented shield was used as the transmit volume coil. The peak RF power in total was 5, 5, and 24 kW (12 kW per port) for ^2^H, ^23^Na, and ^31^P frequencies, respectively. The average power remained well below the SAR levels of 8 W/kg peak and 3 W/kg global based on the SAR simulations. The body coil was driven in quadrature mode at all frequencies.

Two different receive coils were employed. An eight‐channel ^2^H/^31^P receive array with eight transmit‐receive fractioned transmit/receive ^1^H dipole antennas [28] (WaveTronica B.V., Utrecht, the Netherlands) was used in ^2^H/^31^P phantom imaging and ^2^H in vivo abdomen imaging. A quintuple‐tuned receive whole brain coil (^1^H/^19^F/^31^P/^23^Na/^13^C, TeslaDC, Zaltbommel, the Netherlands) [23] was employed in ^23^Na phantom imaging and ^23^Na/^31^P in vivo imaging. Each acquisition set of metabolic imaging started with ^1^H acquisitions to determine the FOV. Then, nominal flip angle series were applied to calculate the corresponding B_1_^+^ field strength during phantom studies. Series included 10°–200° with a step of 10° at ^2^H, ^23^Na, and ^31^P frequencies.

Scan parameters of phantom studies, in vivo studies, and B_1_^+^ maps are detailed in Table 1. In vivo ^2^H FID was obtained from the liver of a healthy male volunteer (49 years old), whereas in vivo ^23^Na FID and ^31^P MRSI were obtained from the brain of a healthy female volunteer (34 years old) with informed consent. B_1_^+^ maps at ^2^H and ^31^P frequencies were acquired using a multi‐nuclei body phantom (3 g/L NaCl, 150 g H_2_KO_4_P, 137 mL D_2_O, 26 L H_2_O) and the eight‐channel ^2^H/^31^P receive array. ^23^Na B_1_^+^ map was acquired using a sphere phantom (6.5 g/L H_2_KO_4_P, 3.4 g/L NaCl, 645 mL H_2_O) and the quintuple‐tuned receive whole brain coil.

**TABLE 1:** The scan parameters of phantom studies, in vivo studies, and B_1_^+^ maps.

Parameters	Phantom			In vivo			B_1_^+^ maps		
	^2^ H	^23^ Na	^31^ P	^2^ H‐FID	^23^ Na‐FID	^31^ P‐MRSI	^2^ H	^23^ Na	^31^ P
TR (ms)	1000	250	1000	2000	250	1000	50 650	50 1200	50 950
TE (ms)	14.12	1.26	2.16	2.56	1.75	2.96	3.9	2.8	1.66
Nominal flip angle (°)	180	45	90	90	45	180	60	60	60
Total scan duration (min:sec)	1:47	3:30	6:59	0:32	6:36	7:15	28:10	09:48	04:12:03
Field of view AP × FH × RL (mm^3^)	400 × 50 × 400	400 × 50 × 400	400 × 50 × 400	300 × 30 × 300	220 × 240 × 240	240 × 240 × 240	300 × 300 × 480	300 × 300 × 480	300 × 300 × 480
Nominal voxel size LR × AP × FH (mm^3^)	12.5 × 12.5 × 50	12.5 × 12.5 × 50	12.5 × 12.5 × 50	300 × 300 × 30	20 × 20 × 20	30 × 30 × 30	20 × 20 × 20	20 × 20 × 20	20 × 20 × 20

### 2.5 Loss Calculation

#### 2.5.1 The Loss of the Copper Conductors

R_endring_ calculated by L7 at ^2^H, ^23^Na, and ^31^P frequencies are 0.02, 0.025, and 0.03 Ω, respectively. Thus, R_endring_ at the ^23^Na and ^31^P frequencies was 1.25‐fold and 1.50‐fold of R_endring_ measured at the ^2^H frequency. R_leg_ calculated through L40 at ^2^H, ^23^Na, and ^31^P frequencies are 0.125, 0.3, and 0.5 Ω, respectively. Hence, R_leg_ at the ^23^Na and ^31^P frequencies was 2.4‐fold and 4.0‐fold of R_leg_ measured at the ^2^H frequency.

#### 2.5.2 The Loss of the Capacitors

**FIGURE 3:**
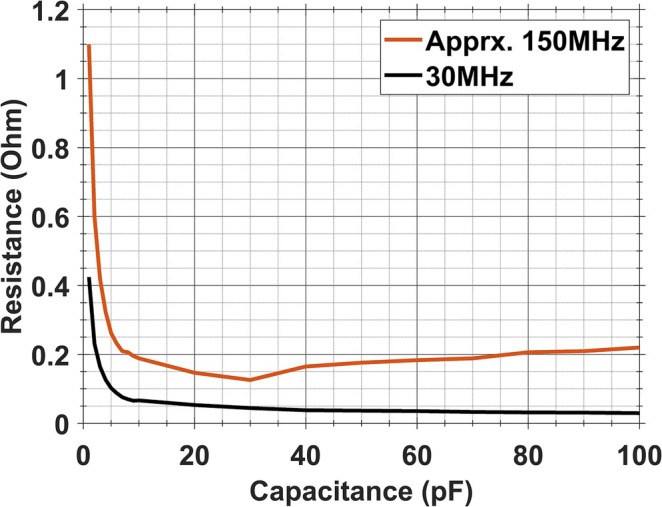
The loss graph of 100E series using the specification document (30 MHz) and the approximated line representing Q‐factors at 150 MHz.

The loss graph from the specification document of the capacitors (Figure 3) at 30 MHz indicates that the loss is maximum at 1 pF, followed by a steep decrease until 10 pF, with relatively identical losses observed between 10 and 100 pF. The loss graph at 150 MHz, derived from the approximate line (Figure 3), demonstrated a consistent trend: capacitances between 1 and 10 pF were more lossy, and losses for those between 10 and 100 pF remained relatively identical. According to the loss graph at 150 MHz, R_10pF_ and R_30pF_ were determined as 0.1 and 0.3 Ω, respectively. The on‐bench experiments conducted at the ^31^P frequency are detailed in Table 2. Specifically, R_10pF_ in L13 and L38 were 0.24 Ω (0.08 Ω*3) and 0.29 Ω, respectively. Additionally, R_30pF_ in L13 and L38 were 0.10 and 0.14 Ω (0.42 Ω / 3), respectively. So, the bench measurements are similar to the extrapolated losses from the vendor specification document.

**TABLE 2:** The on‐bench experiments with loop coils of L13 and L38 to indirectly calculate capacitor loss such as R_10pF_ and R_30pF_.

Loop coils	C (pF)	Q	R (Ω)
L13	30	450	0.10
	10//10//10	560	0.08
L38	10	650	0.29
	30–30–30	440	0.42

#### 2.5.3 The Loss of the Multi‐Tuning Circuits

The loss results of the double‐tuning setups and the triple‐tuning setup are given in Table 3. It is evident that the loss at the ^2^H frequency is comparatively lower than that at higher frequencies such as ^23^Na and ^31^P. Moreover, as the difference between two target frequencies in a double‐tuned circuit decreases, there is a substantial increase in the resulting loss in the circuitry [15] such that the loss at ^31^P frequency on the double‐tuned ^23^Na/^31^P circuit is 35% higher than that on the double‐tuned ^2^H/^31^P circuit. Note that the extra loss when moving from double‐tuned to triple‐tuned is negligible for ^31^P and ^23^Na while adding 0.19 Ω for ^2^H.

**TABLE 3:** Q‐factors and the corresponding losses of the double‐tuned and the triple‐tuned circuits. Results belonging to ^2^H, ^23^Na, and ^31^P are shown with green, red, and purple, respectively.

	Components	Nucleus	Q_on‐bench_	R_measured_ (Ω)
The double‐tuned	C_D1_ = 68 pF L_D1_ = 23 nH C_D2_ = 20 pF	^23^ Na	103	0.69
		^31^ P	138	0.84
	C_D1_ = 33 pF L_D1_ = 68 nH C_D2_ = 56 pF	^2^ H	176	0.21
		^31^ P	175	0.62
The triple‐tuned	C_T1_ = 68 pF L_T1_ = 100 nH C_T2_ = 47 pF L_T2_ = 39 nH C_T3_ = 24 pF	^2^ H	100	0.4
		^23^ Na	140	0.53
		^31^ P	200	0.67

### 2.6 Simulation Results

**FIGURE 4:**
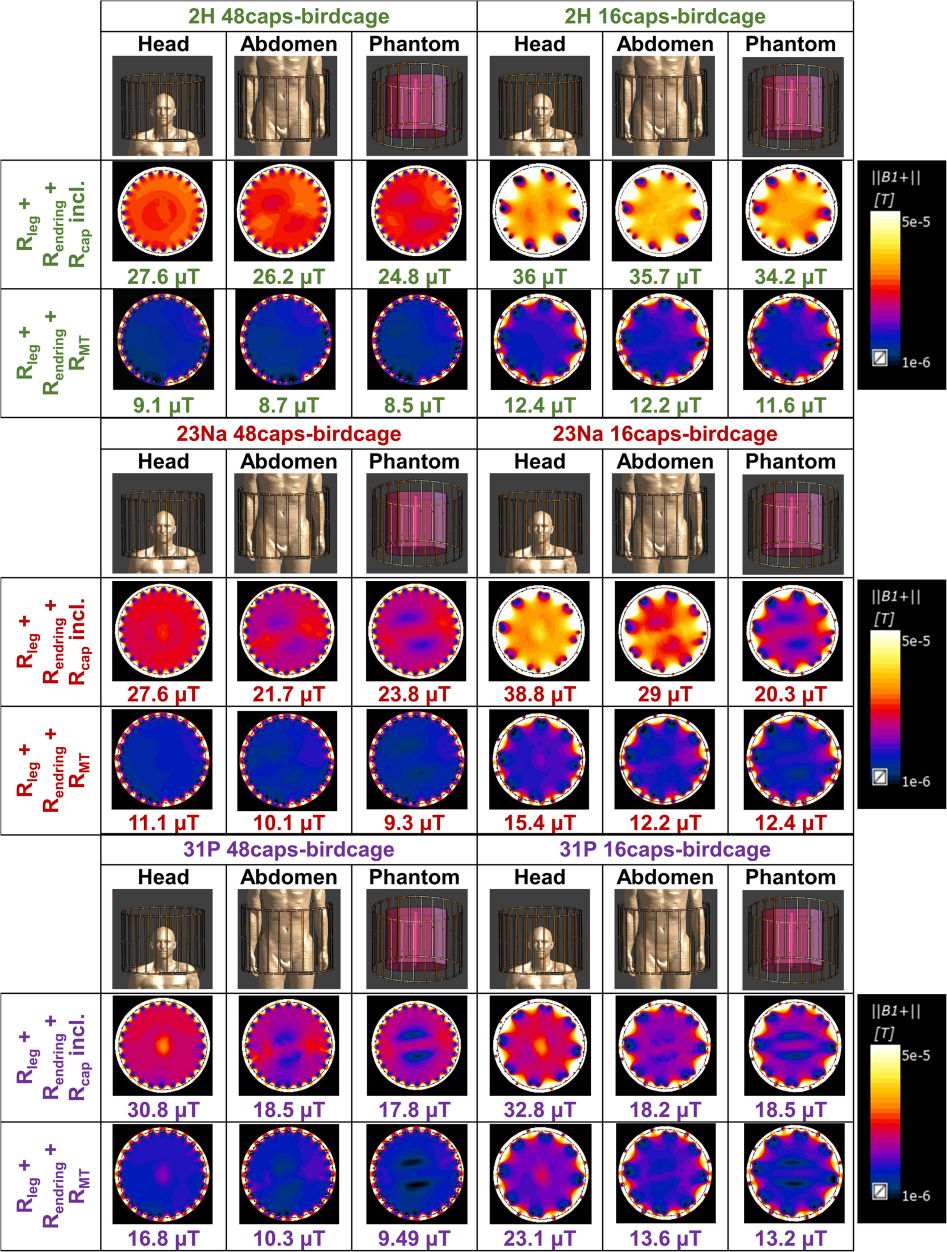
The simulation results of 48caps‐birdcage and 16caps‐birdcage at ^2^H, ^23^Na, and ^31^P frequencies at three different loading cases (head, abdomen, and phantom) and two different loss cases: (1) R_leg_, R_endring_, and R_cap_ included and (2) R_leg_, R_endring_, and R_MT_, representing the single‐tuned loss case and the multi‐tuned loss case, respectively. The B_1_^+^ field strength at the center is written at the bottom of each map. Results belonging to ^2^H, ^23^Na, and ^31^P frequencies are shown in green, red, and purple, respectively.

**FIGURE 5:**
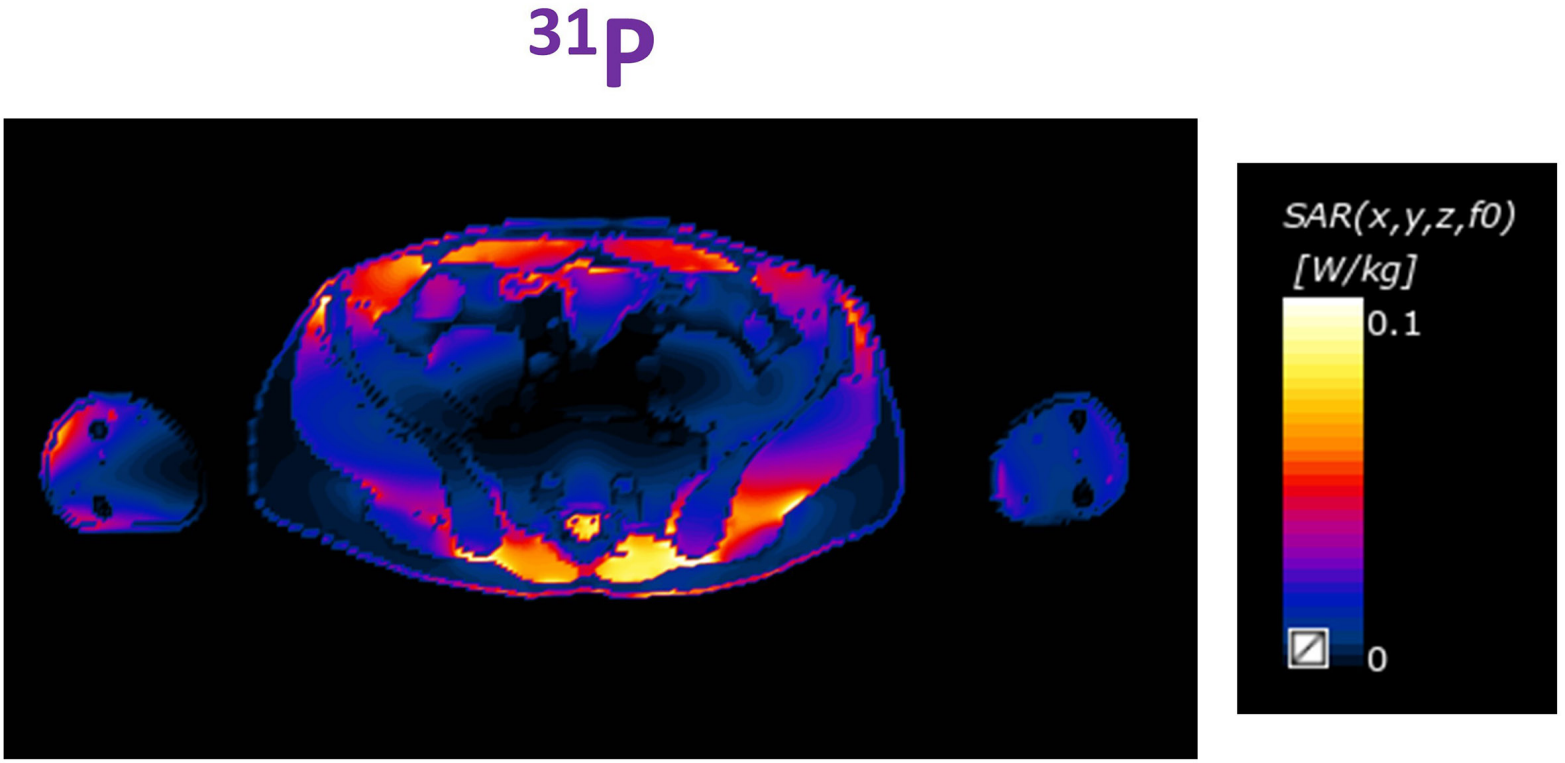
Local SAR computations of the 16caps‐birdcage when loaded with the human model's abdomen at the ^31^P frequency, normalized to 1 W.

The simulated B_1_^+^ field intensity at the center of the 48caps‐birdcage and the 16caps‐birdcage under different loss cases and loading types is given in Figure 4. Local SAR calculations of the 16caps‐birdcage when loaded with the human model's abdomen are given in Figure 5 for all frequencies.

#### 2.6.1 The Single‐Tuned (R_leg_, R_endring_, and R_cap_ Included) Case

The coil loss‐dominated case (head) of both the 48caps‐birdcage and the 16caps‐birdcage yielded the highest B_1_^+^ field intensity for all nuclei compared with the load loss‐dominated cases (body and phantom). However, the B_1_^+^ field intensity in the load loss‐dominated case decreased 5.1%–10.1% in ^2^H 48caps‐birdcage, 0.8%–5.0% in ^2^H 16caps‐birdcage, 13.8%–21.3% in ^23^Na 48caps‐birdcage, 25.3%–47.7% in ^23^Na 16caps‐birdcage, 39.9%–42.2% in ^31^P 48caps‐birdcage, and 43.6%–44.5% in ^31^P 16caps‐birdcage compared with the coil loss‐dominated case. The decreases in the percentage are calculated considering B_1_^+^ values in the coil loss‐dominated case. Consequently, the 16caps‐birdcage provided the highest B_1_^+^ field in this case.

#### 2.6.2 The Multi‐Tuned (R_leg_, R_endring_, and R_MT_) Case

The B_1_^+^ efficiency when comparing the 16caps‐birdcage to the 48caps‐birdcage increased by 36%/39%/38% for ^2^H/^23^Na/^31^P when loaded with the head, 40%/21%/32% for the body, and 36%/33%/39% for the phantom, all assuming a 1 Ω loss for the multi‐tuned circuitry. When corrected for the actual measured resistive loss in the circuitries, this will be a gain of 23%/24%/32% for ^2^H/^23^Na/^31^P.

### 2.7 Bench Measurements

#### 2.7.1 The Double‐Tuned Body Coils

**FIGURE 6:**
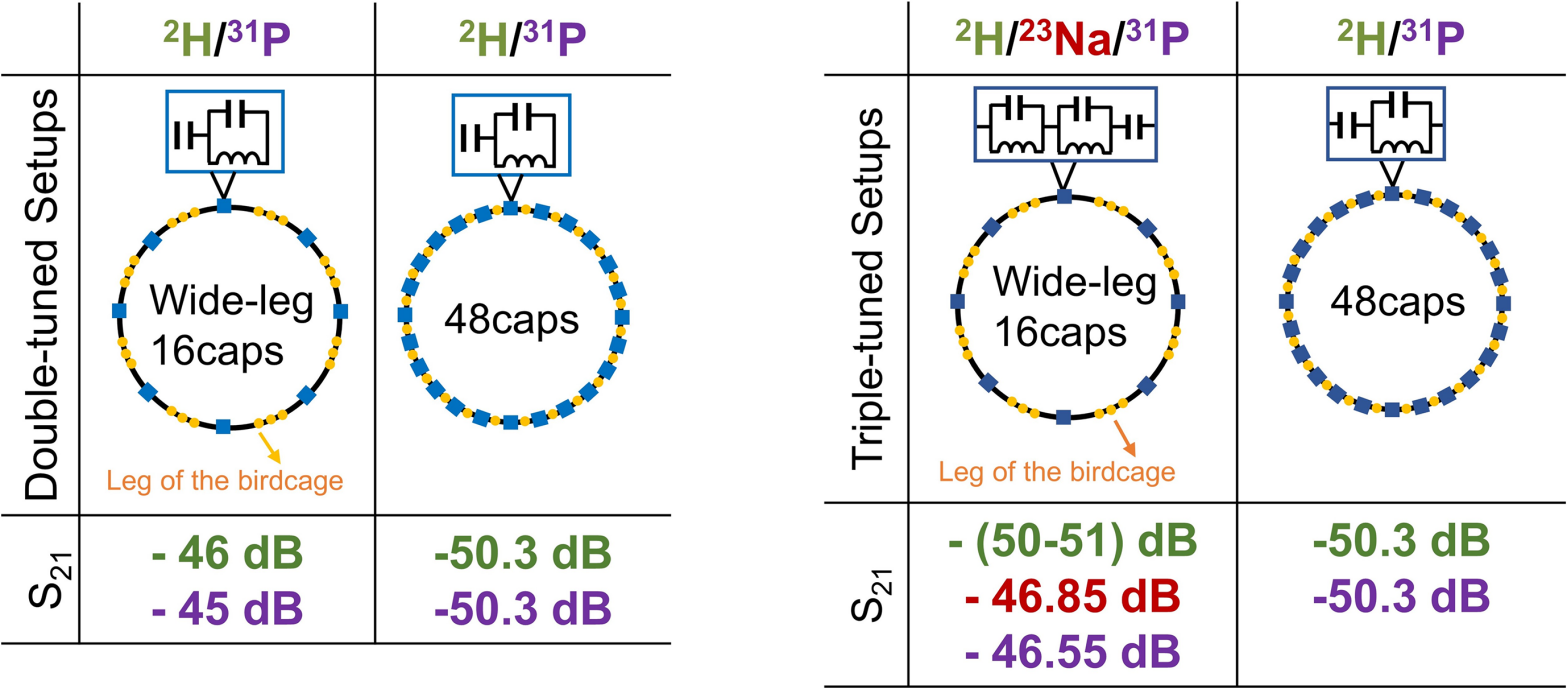
The double‐tuned and the triple‐tuned bench experiments. Measurements belonging to ^2^H, ^23^Na, and ^31^P are shown in green, red, and purple, respectively. Yellow dots indicate the legs of the birdcage design. Blue squares show the tuning elements in the designs, which are double‐tuned circuits in double‐tuned setups, and triple‐tuned circuits in the triple‐tuned setups. The coil efficiency (S_21_) is given for each experiment. The wide‐leg 16caps‐birdcage was double‐tuned at ^2^H/^31^P frequencies and triple‐tuned at ^2^H/^23^Na/^31^P frequencies. The reference double‐tuned body coil is the ^2^H/^31^P 48caps‐birdcage.

The efficiency results of the reference double‐tuned body coil and the double‐tuned ^2^H/^31^P wide‐leg 16caps‐birdcage coil are given in Figure 6. The wide‐leg 16caps‐birdcage provided 4.3 and 5.3 dB gain at ^2^H and ^31^P frequencies, respectively, compared with the reference double‐tuned body coil.

#### 2.7.2 The Triple‐Tuned Wide‐Leg 16caps‐Birdcage

The wide‐leg 16caps‐birdcag design improved the efficiency at the ^23^Na and the ^31^P frequencies by 3.45 and 3.75 dB increase, respectively (Figure 6). On the other hand, the ^2^H efficiency decreased by 4–5 dB back to its original efficiency, which is similar (−0.7 dB until +0.3 dB) to the one in the reference double‐tuned body coil. We concluded that the wide‐leg 16caps‐birdcage provided the optimum efficiency results for all nuclei.

### 2.8 Phantom and In Vivo Imaging

The triple‐tuned wide‐leg 16caps‐birdcage was inserted in the MRI system for the phantom and in vivo experiments. S_11_2H_, S_11_23Na_, and S_11_31P_ were −14.3, −10.4, and −11.6 dB, respectively, whereas S_22_2H_, S_22_23Na_, and S_22_31P_ were −12.0, −8.5, and −11.5 dB, respectively, when the coil was loaded with the body phantom.

**FIGURE 7:**
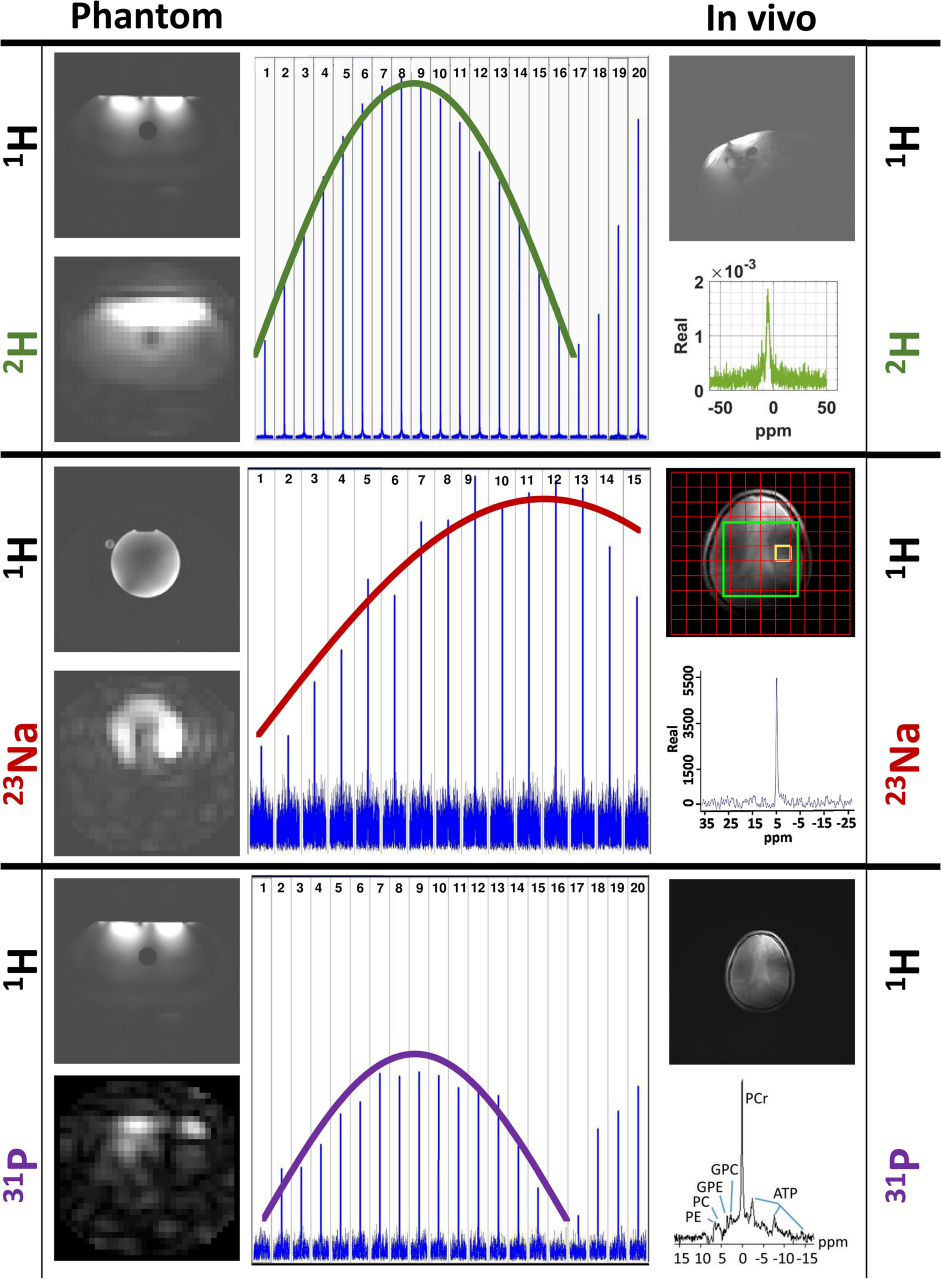
Phantom and in vivo images of all nuclei are given on the left‐hand side and the right‐hand side, respectively. The nominal flip angle series applied for each nucleus during phantom imaging are shown in the middle. In vivo ^2^H FID from the liver, ^23^Na FID from the brain, and ^31^P MRSI from the brain are demonstrated. Results belonging to ^2^H, ^23^Na, and ^31^P are shown with green, red, and purple, respectively.

The corresponding FID signal was obtained from the area where the receive coils were placed. MRSI from ^2^H, ^23^Na, and ^31^P is shown in phantoms as well as the spectra in vivo of the healthy volunteers (Figure 7).

**FIGURE 8:**
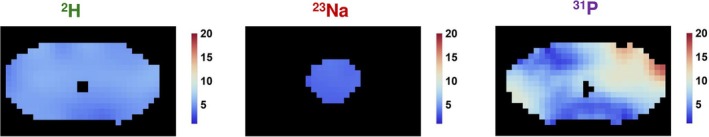
B_1_^+^ maps of the triple‐tuned (^2^H/^23^Na/^31^P) wide‐leg 16caps‐birdcage coil at ^2^H, ^23^Na, and ^31^P frequencies.

At the forward peak power of 5, 5, and 24 kW from the RF amplifier, the calculated B_1_^+^ field strength of ^2^H, ^23^Na, and ^31^P frequency was 5.1, 4.1, and 13.5 μT, respectively, based on the nominal flip angle series (Figure 7). B_1_^+^ maps at ^2^H, ^23^Na, and ^31^P frequencies are demonstrated in Figure 8. ^2^H and ^23^Na B_1_^+^ maps are homogeneous, whereas ^31^P B_1_^+^ field strength varies up to 20 μT throughout the FOV.

## 3 Discussion

This study thoroughly investigated the loss mechanisms of high‐pass multi‐tuned birdcage coils in terms of copper conductor loss, capacitor loss, and tank circuit loss. Figure 4 supports the fact that the tissue loss between 45 and 120 MHz surpasses the coil loss [23, 24]. Apart from the body loss, the capacitor loss on the endrings had a major effect on the efficiency (Figure 4). In most double‐tuned coils, the extra losses are mainly caused by the additional inductor in the tank circuit. However, we noticed significant capacitor losses even in single‐tuned birdcage coils, which only have tuning capacitors and no trap circuits. We found that the losses in Case‐E capacitors vary with their capacitance value. Specifically, capacitors with values between 1 and 10 pF have the highest losses, while those between 10 and 100 pF are more efficient (Figure 3). To achieve the desired double‐tuning or triple‐tuning at specific frequencies, certain capacitor values are required. Therefore, designing the most efficient triple‐tuned setup will become a function of the used capacitors. The examination of capacitor loss highlighted the importance of not overlooking the loss in high‐voltage capacitors, given their substantial loss contribution and dominant influence on coil efficiency. This is in agreement with the work of Kuehne et al. [5] that explored the loss mechanisms at 297 MHz. Overall loss examination has shown that one should include losses in EM simulations specific to the designed birdcage coil to obtain more realistic efficiency results.

Including the losses becomes even more apparent for multi‐tuned circuitries as the effective resistive loss of tank circuits will increase due to partial resonance of electrical currents in these tanks. Our research showed that the loss at the ^31^P frequency in the triple‐tuned circuit is lower than that in the ^23^Na/^31^P double‐tuned circuit. The tank circuit in the double or triple‐tuned designs would give the highest loss due to the relatively higher current density in these tanks compared with single‐tuned designs. Distributing this loss over two tanks (triple‐tuned) rather than one (double‐tuned) would give a lower current density and voltage accumulation in each tank circuit. As the power loss goes quadratic with voltage or current and the distribution over multiple circuits most likely goes more linear than quadratic, the overall power balance may become more favorable.

The key to enhancing multi‐tuned birdcage efficiencies lies in minimizing the total loss on the endrings. Hence, increasing the number of legs to achieve a homogenous magnetic field and thus increase the number of capacitors and multi‐tuned circuitries could hamper the efficiency of multi‐tuned birdcages. Consequently, the conventional approach of using the same number of multi‐tuned circuits as the number of legs should be avoided. We therefore reduced the number of circuits on the endrings from 48 to 16. Indeed, the presence of additional legs on the wide‐leg 16caps‐birdcage did contribute to efficiency improvement as inspired by the multiple parallel legs approach of Xu et al. [27]. Their approach aimed at reducing the loss of the single‐tuned birdcage designs by splitting the current flowing through the legs and therefore decreasing the loss on the legs, while our approach aimed to minimize the loss in the multi‐tuned circuits.

Our study is subject to certain limitations. Firstly, there is a tradeoff between 48caps‐birdcage and 16caps‐birdcage in terms of B_1_^+^ inhomogeneity and B_1_^+^ efficiency. While no significant inhomogeneity is observed regarding the loading type in ^2^H 48caps‐birdcage (Figure 4), mild inhomogeneities in the B_1_^+^ field begin to occur at ^23^Na 48caps‐birdcage and ^31^P 48caps‐birdcage in abdomen and phantom loading. Recalling the wavelength in tissue ($\lambda_{r}=\frac{c_{0}}{\sqrt{\varepsilon_{r}}\cdot f}$), where $\lambda_{r}$ is the wavelength in medium, $c_{0}$ is speed of light in free space, $\varepsilon_{r}$ is the permittivity of the medium, and *f* is the frequency, the wavelength for $\varepsilon_{r}=74$ at the ^2^H, ^23^Na, and the ^31^P frequency is 76, 44, and 29 cm, respectively. Hence, inhomogeneities result from the relatively short wavelength of ^23^Na and ^31^P frequencies in typical media. Indeed, ^31^P B_1_^+^ maps from the body phantom that has a relatively high dielectric permittivity (Figure 8) validates its simulated B_1_^+^ field in terms of inhomogeneity.

Alternative strategies have been proposed to use smaller‐sized X‐nuclei body coils that can be positioned on the MRI bed and guided with the subject inside the MRI [29]. Our setup facilitates a relatively uniform B_1_^+^ field to excite ^2^H, ^23^Na, and ^31^P spins throughout the human body without compromising space for the subject to be scanned. While care must be given to improve matching and reduce gradient eddy currents, our concept may be an attractive setup for facilitating metabolic MRI throughout the body. Designing a triple‐tuned ^2^H/^23^Na/^31^P receive body array that would accompany the triple‐tuned ^2^H/^23^Na/^31^P body coil could enable whole‐body metabolic MRI acquisition in a single session.

## Supporting information


**Figure S1:** Implemented birdcages on the bench. (a) The reference double‐tuned (^2^H/^31^P) body coil with the segmented shield. (b) The double‐tuned (^23^Na/^31^P) 16caps‐birdcage with the continuous mesh shield. (c) The triple‐tuned (^2^H/^23^Na/^31^P) wide 8‐leg birdcage with the improved segmented shield. (d) The enlarged view of the single leg of the wide 8‐leg birdcage to demonstrate three closely placed rods.

**Figure S2:** The double‐tuned and the triple‐tuned bench experiments. Measurements belonging to ^2^H, ^23^Na, and ^31^P are shown with green, red, and purple, respectively. Yellow dots indicate the legs of the birdcage design. Blue squares show the tuning elements in the designs, which are double‐tuned circuits in double‐tuned setups and triple‐tuned circuits in the triple‐tuned setups. The coil efficiency (S_21_) and the reflection coefficient value (S_11_) are given for each experiment. The 16caps‐birdcage (16caps), the classical 8‐leg, and the wide 8‐leg birdcages were double‐tuned at ^2^H/^31^P frequencies, whereas the 16caps‐birdcage was double‐tuned at ^23^Na/^31^P frequencies. The reference double‐tuned body coil is the ^2^H/^31^P 48caps‐birdcage.

**Figure S3:** The triple‐matching design for Port 1 and Port 2. The triple‐tuned circuit composed of C_M1_, C_M2_, L_M1_, C_M2_, L_M2_ provides three different capacitance, 70, 30, and 13 pF at ^2^H, ^23^Na, and ^31^P frequencies, respectively.

## Data Availability

The data that support the findings of this study are available upon reasonable request from the corresponding author. The data are not publicly available due to privacy or ethical restrictions.
