# Supplementary figures and images for: An Integrated B1 + Efficient Triple‐Tuned (2H/23Na/31P) Body Coil at 7T
Source: NMR Biomed. 2025 Aug 7;38(9):e70118. doi: 10.1002/nbm.70118 (PMC12331556; doi:10.1002/nbm.70118)

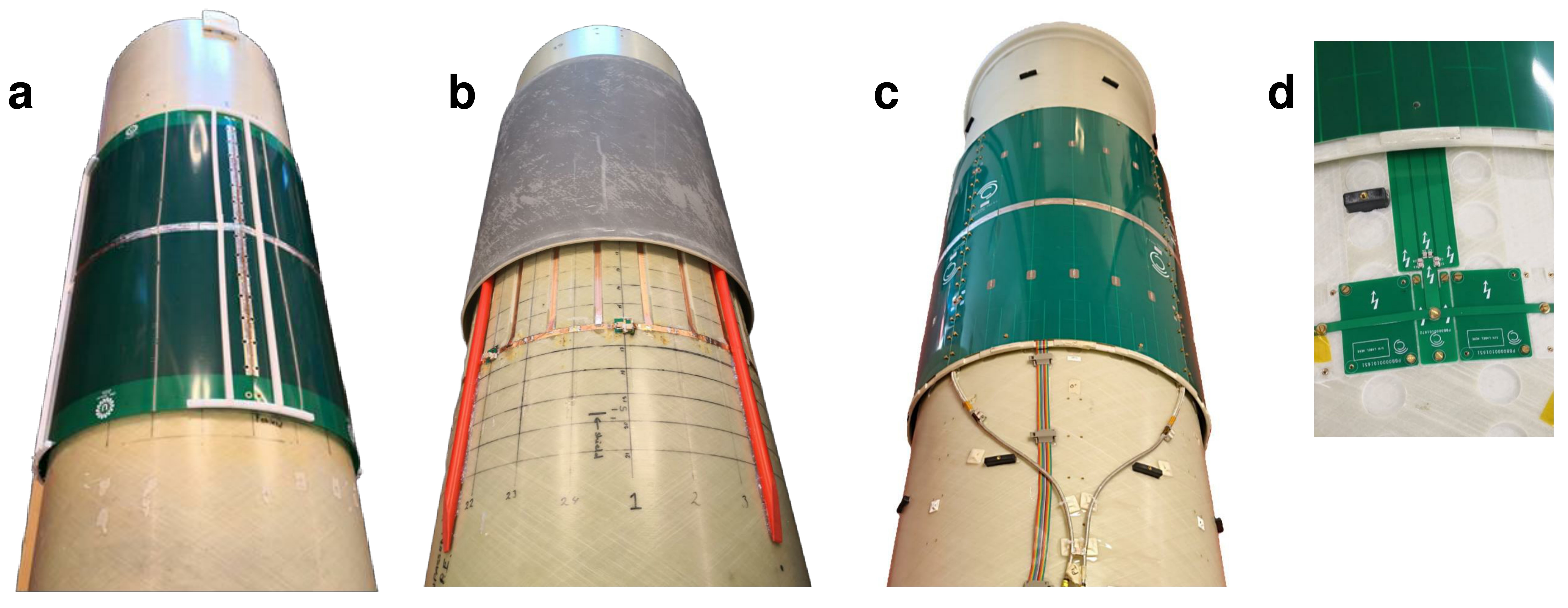

Supplement: Supplementary file 1 — Figure S1: Implemented birdcages on the bench. (a) The reference double‐tuned (2H/31P) body coil with the segmented shield. (b) The double‐tuned (23Na/31P) 16caps‐birdcage with the continuous mesh shield. (c) The triple‐tuned (2H/23Na/31P) wide 8‐leg birdcage with the improved segmented shield. (d) The enlarged view of the single leg of the wide 8‐leg birdcage to demonstrate three closely placed rods. [file NBM-38-e70118-s002.jpg]

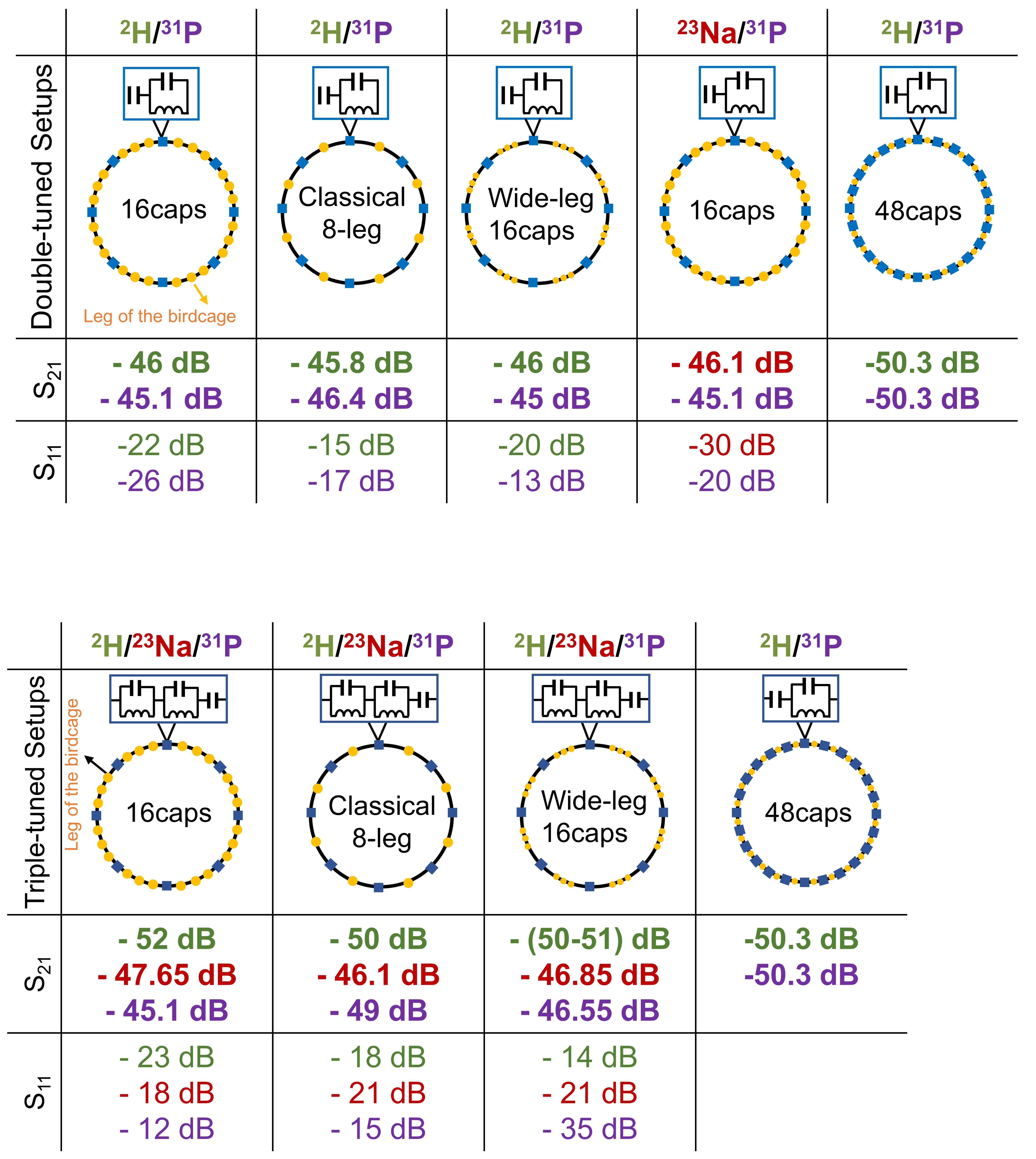

Supplement: Supplementary file 2 — Figure S2: The double‐tuned and the triple‐tuned bench experiments. Measurements belonging to 2H, 23Na, and 31P are shown with green, red, and purple, respectively. Yellow dots indicate the legs of the birdcage design. Blue squares show the tuning elements in the designs, which are double‐tuned circuits in double‐tuned setups and triple‐tuned circuits in the triple‐tuned setups. The coil efficiency (S21) and the reflection coefficient value (S11) are given for each experiment. The 16caps‐birdcage (16caps), the classical 8‐leg, and the wide 8‐leg birdcages were double‐tuned at 2H/31P frequencies, whereas the 16caps‐birdcage was double‐tuned at 23Na/31P frequencies. The reference double‐tuned body coil is the 2H/31P 48caps‐birdcage. [file NBM-38-e70118-s001.jpg]

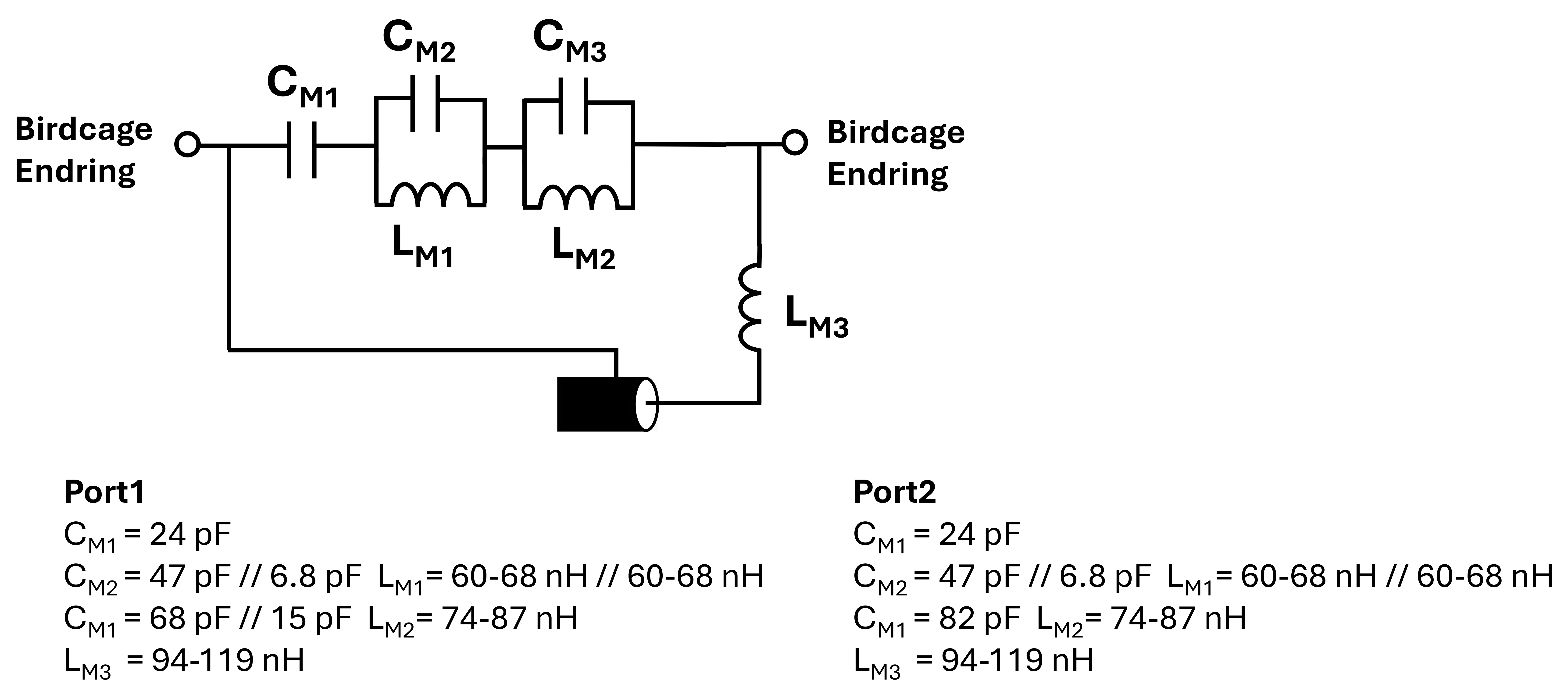

Supplement: Supplementary file 3 — Figure S3: The triple‐matching design for Port 1 and Port 2. The triple‐tuned circuit composed of CM1, CM2, LM1, CM2, LM2 provides three different capacitance, 70, 30, and 13 pF at 2H, 23Na, and 31P frequencies, respectively. [file NBM-38-e70118-s003.png]
